# Systematic Analysis of Expression and Prognostic Values of Lysyl Oxidase Family in Gastric Cancer

**DOI:** 10.3389/fgene.2021.760534

**Published:** 2022-01-20

**Authors:** Li Wang, Shan Cao, Rujun Zhai, Yang Zhao, Guodong Song

**Affiliations:** ^1^ Department of Gastrointestinal Surgery, The Second Hospital of Tianjin Medical University, Tianjin, China; ^2^ Department of Respiratory, The Second Hospital of Tianjin Medical University, Tianjin, China; ^3^ Radiology Department, The Second Hospital of Tianjin Medical University, Tianjin, China

**Keywords:** gastric cancer, LOX family, bioinformatics analysis, biomarker, prognosis

## Abstract

**Background:** Gastric cancer (GC) remains the fifth most commonly diagnosed malignancy worldwide, with a poor prognosis. The lysyl oxidase (LOX) family, a type of secreted copper-dependent amine oxidases, is comprised of LOX and four LOX-like (LOXL) 1–4 isoforms and has been reported to be dysregulated in a number of different type cancers. However, the diverse expression patterns and prognostic values of LOX family in GC have yet to be systematically analyzed.

**Methods:** ONCOMINE, GEPIA, UALCAN, Kaplan–Meier Plotter, LOGpc, cBioPortal, GeneMANIA and Metascape databases were utilized in this study to analyze the expression, prognostic values, mutations and functional networks of LOX family in GC.

**Results:** The mRNA expression levels of LOX, LOXL1 and LOXL2 were significantly higher in GC, the expression level of LOXL3 was contrary in different databases, while the expression level of LOXL4 made no difference; the expression levels of LOX, LOXL1 and LOXL3 were higher in stages 2–4 than that of normal tissues and stage 1, while the mRNA level of LOXL2 in stage 1–4 was higher than normal tissues; patients with high expression of LOX and LOXL 2-4 had poor OS; the genes correlated with LOX and LOXL2 were enriched in extracellular matrix organization, vasculature development and skeletal system development.

**Conclusion:** Our results indicated that the LOX family, especially LOX and LOXL2, might play an important role in GC oncogenesis, and they may become biomarkers for predicting tumor prognosis and potential targets for tumor therapy.

## Introduction

Even though declines in (GC)incidence and mortality rates have been observed consistently across world regions, GC remains the fifth most commonly diagnosed malignancy worldwide, with over 1 million estimated new cases in 2018 (Bray et al., 2018). However, due to its advanced-stage diagnosis, excess mortality from this cancer is high, making GC the third most common cause of cancer related death with 784,000 deaths globally (Bray et al., 2018). Despite major advances in understanding the epidemiology, pathology, and molecular mechanisms of GC and in implementing emerging therapeutic options such as targeted and immune-based therapies, not all patients respond to existing molecularly targeted agents developed for certain acknowledged biomarkers (Chau, 2017). As a result, it is of great importance to further identify novel diagnostic and prognostic biomarkers in terms of the biological complexity, poor prognosis and high reoccurrence of GC (Kang et al., 2018).

The extracellular matrix (ECM) is a complex network of secreted molecules, the function of which is to program cell behavior including supporting cell adhesion, survival and migration. The remodeling of the ECM in cancer plays an important role in controlling the progression of disease and influences cell growth, motility and survival (Yamauchi et al., 2018a). The lysyl oxidase (LOX) family, which are well known as ECM-modifying proteins, they participate in the crosslinking of collagens and elastin in the ECM, promoting its maturation (Yamauchi et al., 2018b; Chitty et al., 2019). The LOX family, a type of secreted copper-dependent amine oxidases, is comprised of five homologous members: LOX and lysyl oxidase-like proteins 1–4 (LOXL1, LOXL2, LOXL3 and LOXL4) (Molnar et al., 2003). Structurally, these members are all characterized by a highly conserved C-terminal domain and a variable N-terminal domain. The composition of C-terminal domain contains copper binding domain, amino acid residues forming lysine tryosylquinone (LTQ), cofactor formation, and a cytokine receptor-like (CRL) domain (Wang et al., 2016). The pro-domains are existed in the N-terminal region of LOX and LOXL1, whereas four scavenger-receptor cysteine-rich (SRCR) domains in the N-terminal are observed in LOXL2-4 (Xiao and Ge, 2012). Mature active forms of LOX and LOXL1 are obtained through a specific cleavage process induced by bone morphogenetic protein 1 (BMP-1), whereas LOXL2, LOXL3, and LOXL4 do not require this cleavage process to mature. In particular, a pre-pro-LOX protein is encoded by LOX mRNA and converted to the inactive LOX preprotein (pro-LOX) in the cytoplasm. The Pro-LOX protein is further cleaved by BMP-1 to form an active LOX with the LOX propeptide (LOX-PP) to perform its function (Wang et al., 2016). In healthy tissue, the synthesis of the LOX family is tightly regulated to control the amount of active LOX family members present. While the LOX family has been reported to be dysregulated in a number of different type cancers (Li et al., 2015; Salvador et al., 2017; Shao et al., 2019; Zeltz et al., 2019; Hu L. et al., 2020). The changes in LOX family member regulation, expression and subsequently enzymatic activity are therefore important factors in cancer progression (Setargew et al., 2021). The LOX family of enzymes may be favorable targets for anti-stromal therapeutics due to their importance in cancer development and progression when compared to healthy state ECM (Setargew et al., 2021). Additionally, highly LOX family expressing tumors have increased LOX family levels detectable in plasma (Rachman-Tzemah et al., 2017), and thus indicate the potential to be used as tumor serum markers.

The dysregulated expression level of LOX family and their relationship with clinicopathological features and prognosis have been partly reported in human GC. With the revolutionized development of microarray and bioinformatic technology, we conducted this systematical study using the data from The Cancer Genome Atlas (TCGA) and other versatile public databases to analyze the expression levels, mutations, functional networks and prognostic values of different LOX in GC, so as to reveal potential diagnostic, therapeutic, and prognostic targets for GC, and the results in different databases were verified with each other to make the results more convincible.

## Materials and Methods

### Oncomine Database

The mRNA expression levels of LOX family in various cancers and their normal tissue counterparts were analyzed using the Oncomine database (http://www.oncomine.org/) (Rhodes et al., 2007). A *p*-value of 0.001, a fold change of 2, and a gene rank in the top 10% were set as the significance thresholds. The *p* value was calculated using the Student’s *t*-test.

### GEPIA Database

GEPIA (http://gepia2.cancer-pku.cn/) is a gene expression analysis web which contains 9,736 tumors and 8,587 normal samples from the TCGA and the Genotype-Tissue Expression (GTEx) project (Tang et al., 2019). Here we used GEPIA to compare the expression levels between TCGA cancer and matched TCGA normal and GTEx normal. The results were expressed as boxplots, and the cutoff criteria were set as *p* < 0.01 and |Log2FC| > 1.

### UALCAN

UALCAN (http://ualcan.path.uab.edu/) is a comprehensive, user-friendly, and interactive web resource for analyzing cancer OMICS data (Chandrashekar et al., 2017). In this study, we used UALCAN to compare the expression levels of LOX family and their relationship with tumor stages. Student’s *t*-test was used to generate a *p*-value and the *p*-value cutoff was 0.05.

### Survival Analysis

We used The Kaplan Meier plotter (http://kmplot.com/analysis/) and LOGpc (http://bioinfo.henu.edu.cn/DatabaseList.jsp) to evaluate the prognostic value of LOX family mRNA expression in which cancer patients were split into high and low expression group based on median values of mRNA expression and validated by K-M survival curves. The Kaplan Meier plotter is capable to assess the effect of 54 k genes (mRNA, miRNA, protein) on survival in 21 cancer types including breast, ovarian, lung and GC (Szász et al., 2016). LOGpc (Long-term Outcome and Gene Expression Profiling Database of pan-cancers) encompasses 209 expression datasets, provides 13 types of survival terms for 31,310 patients of 27 distinct malignancies (Liu et al., 2018). The log-rank test was used for computing *p*-value, with the hazard ratio (HR) and 95% confidence intervals (CI), and *p* < 0.05 was regarded as significant.

### cBioPortal

The cBioPortal (https://www.cbioportal.org/) for Cancer Genomics provides visualization, analysis and download of large-scale cancer genomics data sets (Cerami et al., 2012). The stomach adenocarcinoma (TCGA, Firehose Legacy) dataset was selected to figure out the alterations of LOX family. We also estimated the correlations of each LOX family by analyzing their mRNA expression (RNA Seq V2 RSEM), and then the Spearman correlation coefficient was put into Microsoft Excel 2016 to draw the heat maps. Besides, genes with the highest expression correlation with each LOX protein were generated by cBioPortal, and the top 50 co-expressed genes with highest Spearman correlation score were included in the following functional enrichment analysis.

### GeneMANIA Database

GeneMANIA (https://genemania.org/) is actively developed at the University of Toronto, in the Donnelly Centre for Cellular and Biomolecular Research, in the labs of Gary Bader and Quaid Morris, and it can find other genes that are related to a set of input genes, using a very large set of functional association data (Warde-Farley et al., 2010). GeneMANIA constructed protein–protein interaction (PPI) networks in terms of physical interaction, coexpression, predicted, colocalization, common pathway, genetic interaction, and shared protein domains. In this study, GeneMANIA was used to generate and analyze gene co-expression network.

### Metascape

We used the Metascape web (http://metascape.org) (Zhou et al., 2019) to perform functional enrichment analysis by using the top 50 co-expressed genes of LOX family. The functional process and pathway, following the default, included Canonical Pathway (MSigDB), Hallmark Gene Sets (MSigDB), Kyoto Encyclopedia of Genes and Genomes (KEGG) Pathway and Gene Oncology (GO).

## Results

### Transcriptional Levels of LOX Family in GC and Other Cancers

**FIGURE 1:**
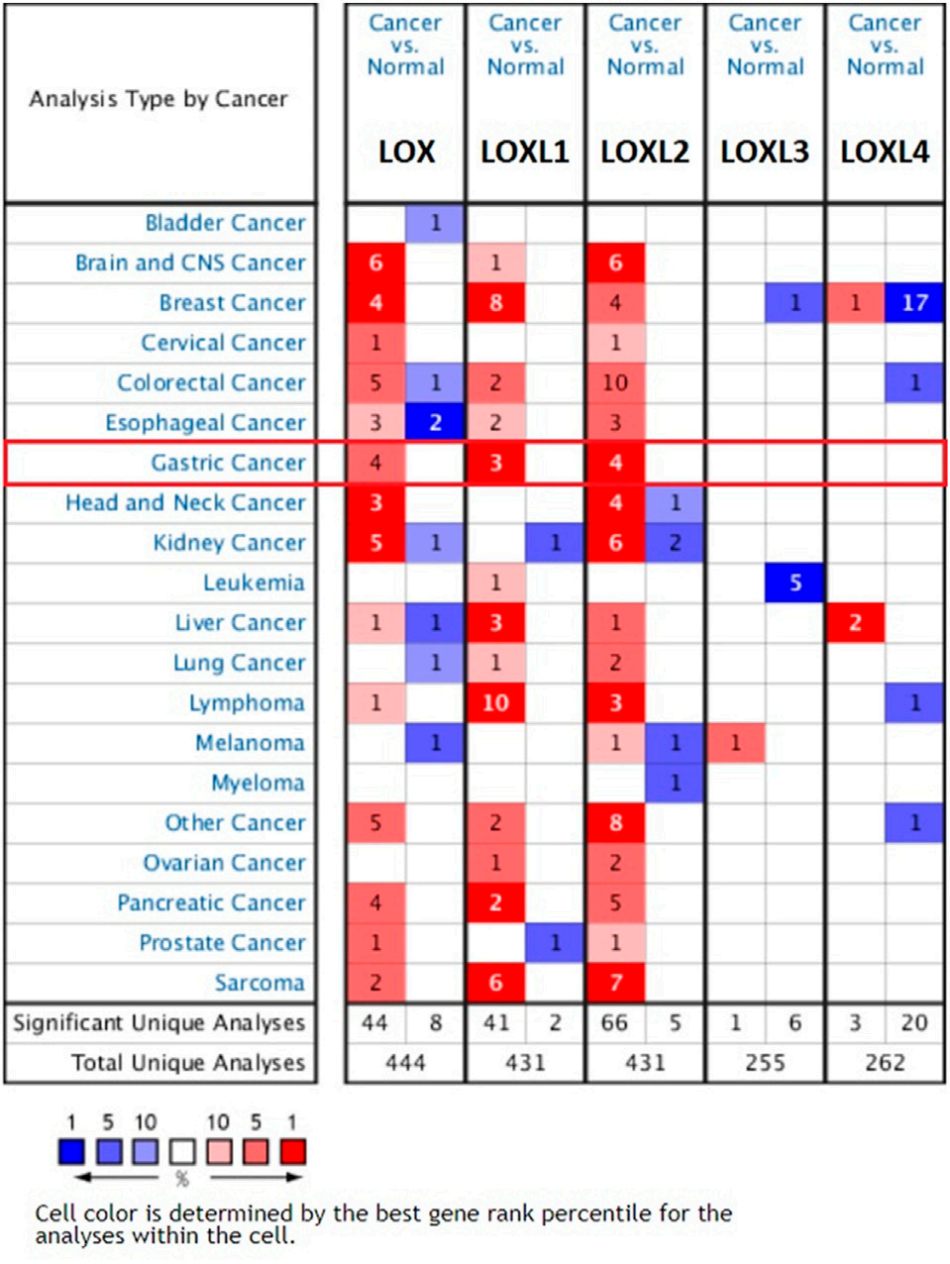
Transcription levels of LOX family in different cancer types (Oncomine).

The transcription level differences of LOX family between tumor and normal tissues were analyzed in multiple cancer types using the Oncomine database. As shown in Figure 1, the expressions of LOX, LOXL1 and LOXL2 have been up-regulated in most of the studied tumors, while LOXL3 and LOXL4 only have differences in expression in a small number of tumors.

The mRNA expression level of LOX was significantly up-regulated in patients with GC in 4 analyses out of 23 in 2 datasets out of 7. Chen gastric statistics (Chen et al., 2003) indicated that LOX is overexpressed in gastric intestinal adenocarcinoma compared with gastric normal tissue with a fold change of 2.312, diffuse gastric adenocarcinoma with a fold change of 2.004, and gastric mixed adenocarcinoma with a fold change of 3.232 (Table 1). Wang gastric analysis (Wang et al., 2012) revealed that LOX is upregulated in GCwith a fold change of 2.287 (Table 1). The transcriptional level of LOXL1 was significantly up-regulated in GC in 3 analyses out of 23 in 3 datasets out of 7. In Chen’s (Chen et al., 2003) dataset, the expression of LOXL1 was 2.077 times higher in gastric mixed adenocarcinoma than normal tissues (Table 1). In Wang’s dataset (Wang et al., 2012), the expression of LOXL1 was 2.083 times higher in GC tissues than normal tissues (Table 1). In DErrico’s dataset (D'Errico et al., 2009), the expression of LOXL1 was 2.192 times higher in gastric mixed adenocarcinoma than normal tissues (Table 1). Upregulation of LOXL2 was observed in 4 analyses in GC tissues compared with normal tissues, with a fold change of 2.118 in Cui’s dataset (Cui et al., 2011), a fold change of 2.424 in Wang’s dataset, a fold change of 2.039 in Chen’s (Chen et al., 2003) dataset, and a fold change of 2.681 in DErrico’s dataset (D'Errico et al., 2009) respectively (Table 1). The analyses of Oncomine database showed no difference in transcriptional levels of LOXL3 and LOXL4 in GC (Figure 1).

**TABLE1:** Remarkable changes of LOX family expression in transcription level between GC and normal gastric tissues (ONCOMINE).

Genes	Type of GC versus normal	Fold change	*p* value	t value	References
LOX	Gastric intestinal type adenocarcinoma	2.312	8.32E-14	9.498	Chen gastric statistics
	Diffuse gastric adenocarcinoma	2.004	1.56E-5	5.536	Chen gastric statistics
	Gastric mixed adenocarcinoma	3.232	1.79E-4	4.679	Chen gastric statistics
	Gastric cancer	2.287	3.13E-4	4.231	Wang gastric statistics
LOXL1	Gastric mixed adenocarcinoma	2.077	5.58E-6	7.664	Chen gastric statistics
	Gastric cancer	2.083	3.47E-5	4.767	Wang gastric statistics
	Gastric mixed adenocarcinoma	2.192	3.75E-5	7.558	DErrico gastric statistics
LOXL2	Gastric cancer	2.118	7.91E-14	8.084	Cui gastric statistics
	Gastric cancer	2.424	2.93E-5	4.838	Wang gastric statistics
	Diffuse gastric adenocarcinoma	2.039	6.41E-5	4.880	Chen gastric statistics
	Gastric intestinal type adenocarcinoma	2.681	2.19E-9	7.165	DErrico gastric statistics

**FIGURE 2:**
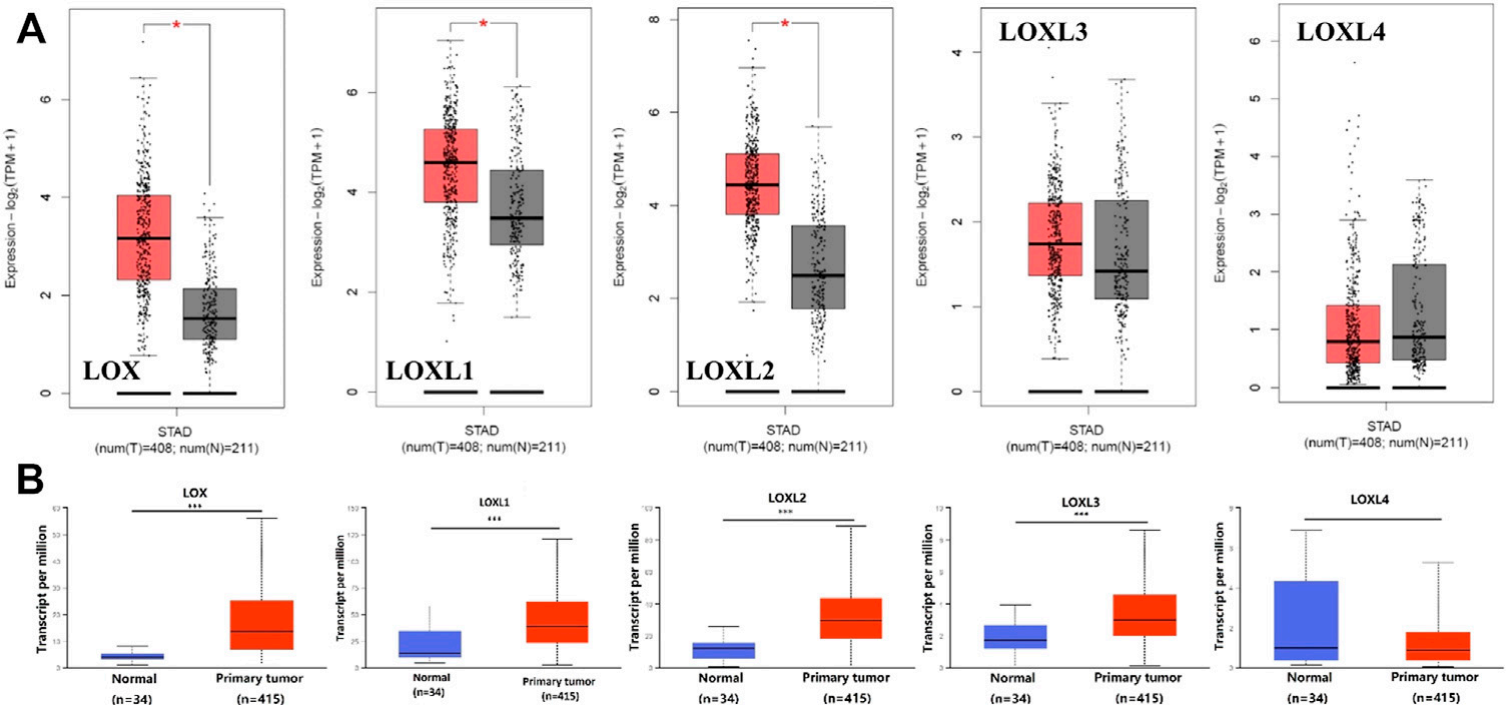
Boxplot showing the expression levels of LOX family inGC. **(A)** GEPIA analysis. The number of normal samples are 211 (grey box), and number of primary tumor samples are 408 (red box), red star means *p* < 0.01; **(B)** UALCAN analysis. The number of normal samples are 34 (blue box), and number of primary tumor samples are 415 (red box) ***p < 0.001.

Furthermore, we used GEPIA to compare the mRNA expression of LOX family between 408 TCGA GC and 211 matched TCGA normal and GTEx normal, used UALCAN to compare the expression levels of LOX family between 415 TCGA GC and 34 TCGA normal. The GEPIA analyses showed that LOX/LOXL1/LOXL2 were higher in GC than in normal tissues (Figure 2A). The results of UALCAN indicated that LOX/LOXL1/LOXL2/LOXL3 were over-expressed in GC (Figure 2B).

### Relationship Between the mRNA Levels of LOX Family and the Cancer Stages of GC

**FIGURE 3:**
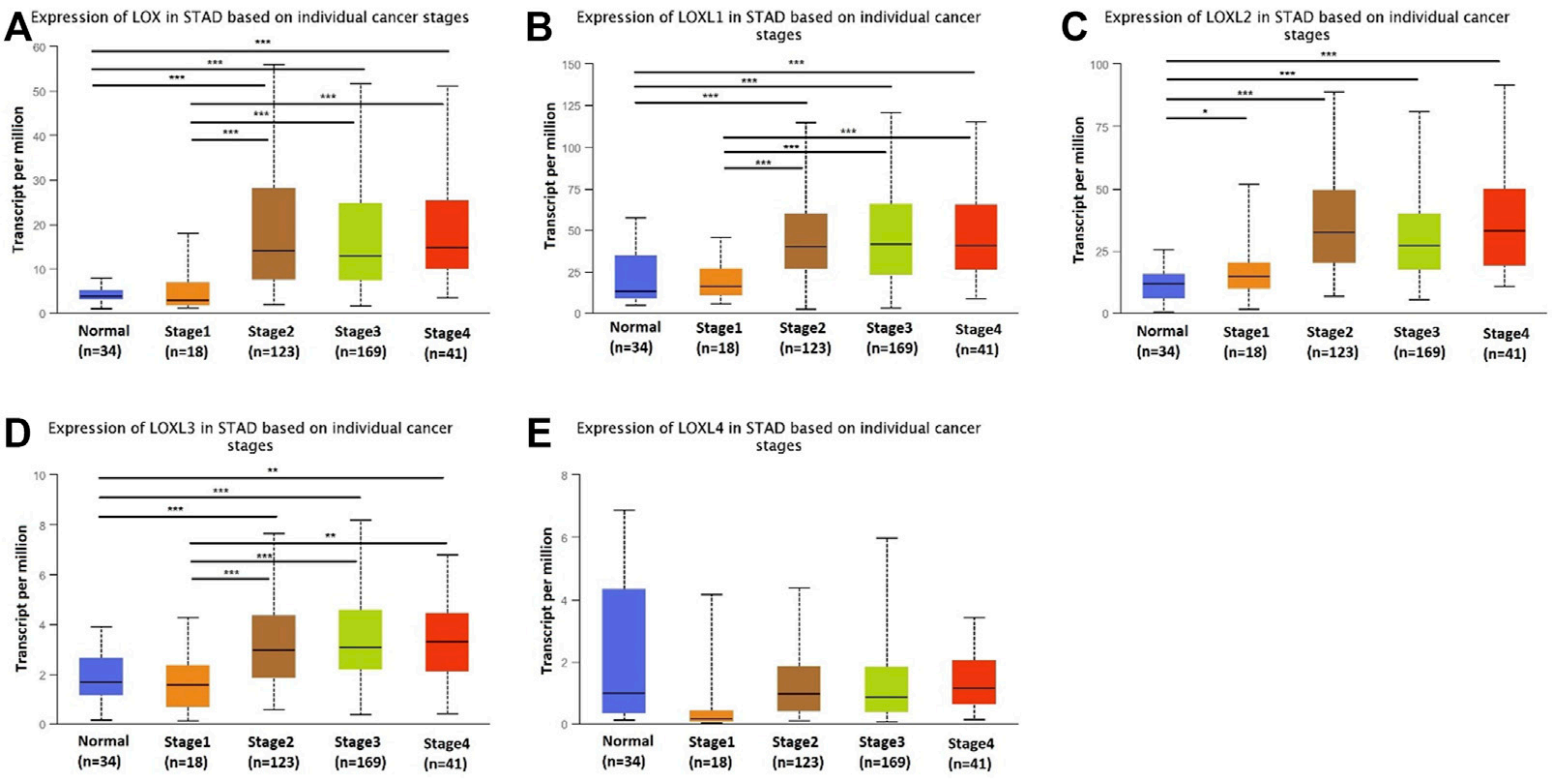
Transcription levels of LOX family in different stage of patients with GC. LOX **(A)**, LOXL1 **(B)**, LOXL2 **(C)**, and LOXL3 **(D)** groups significantly varied, whereas LOXL4 **(E)** groups did not significantly differ. (UALCAN, ***p < 0.001, **p < 0.01, *p < 0.05).

Next, we analyzed the relationship between the mRNA expression of different LOX family members with patients’ individual cancer stages of GC patients by using UALCAN. LOX, LOXL1, LOXL2 and LOXL3 groups significantly varied, whereas LOXL4 groups did not significantly differ (Figure 3). According to clinical stages, the mRNA level of LOX in stage 2–4 was higher than normal tissues and stage 1, while there is no difference between stage 2–4 (Figure 3A). The similar result was found in expression of LOXL1 and LOXL3 (Figures 3B,D). The mRNA level of LOXL2 in stage 1–4 was higher than normal tissues and highest expression was found in stage 2 tissues (Figure 3C). There was no difference in expression of LOXL4 among different stages (Figure 3E).

### Prognostic Values of LOX Family in GC

**FIGURE 4:**
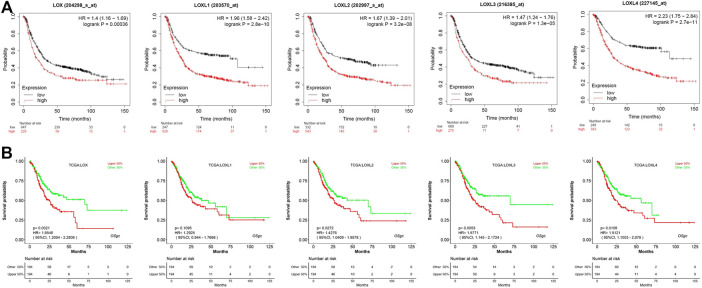
Prognostic feature of mRNA expression of distinct LOX family members in GC patients. **(A)** OS of Kaplan–Meier plotter revealed that all of LOX family members were significantly associated with the OS (*p* < 0.05); **(B)** OS of LOGpc indicated that LOX, LOXL2-4 mRNA expression were associated with OS (*p* < 0.05).

We further explored the prognostic values of LOX family in patients with GC by using the Kaplan–Meier Plotter database and LOGpc database. We separated all GC patients into two groups (high vs. low) based on median expression values for each LOX protein across all GC samples and compared overall survival (OS) between the two groups. The Kaplan-Meier curve and log rank test analyses revealed that all of LOX family members were significantly associated with the OS (*p* < 0.05) in patients with GC (Figure 4A). Meanwhile, the results of LOGpc analyses indicated that the increased LOX, LOXL2-4 mRNA expression were associated with low OS (*p* < 0.05) in patients with GC, but the expression of LOXL1 had no correction with prognosis (*p* > 0.05) in patients with GC (Figure 4B).

### Genetic Mutations and PPI Network of LOX Family

**FIGURE 5:**
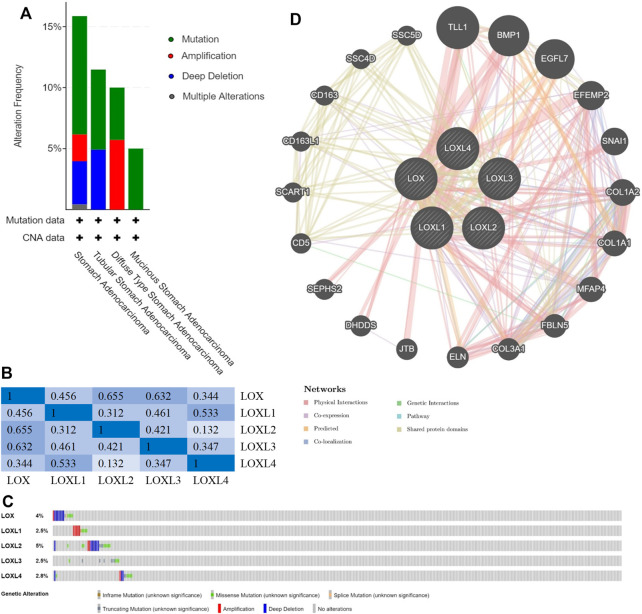
Genomic alterations (cBioPortal) and network (GeneMANIA) of LOX family inGC. **(A)** Distribution of LOX family genomic alterations according to cancer type. **(B)** Correlations of different LOX family members with each other inGC. **(C)** OncoPrint of LOX family alterations inGC. **(D)** Network of the 20 most frequently altered neighboring genes of LOX family.

We analyzed the types and frequency of LOX Family alterations in a cohort of GC patients using cBioPortal. The LOX family were altered in 55 (14%) samples of 393 patients with stomach adenocarcinoma (Figure 5A). We also calculated the correlations of LOX family with each other by analyzing their mRNA expressions (RNA sequencing (RNA seq V2 RSEM)) via the cBioPortal online tool for stomach adenocarcinoma (TCGA, Firehose Legacy) and Pearson’s correction was included. The results indicated significant and positive correlations in the following pairs: LOX and LOXL2, LOX and LOXL3, LOXL1 and LOXL4 (Figure 5B). The mutation rates of LOX, LOXL1, LOXL2, LOXL3 and LOXL4 were 4, 2.5, 5, 2.5 and 2.8%, respectively (Figure 5C).

Next, we used Gene-MANIA to construct a PPI network for LOX family, and the result is shown in Figure 5D. The most top 20 related genes are as follows: TLL1, BMP1, EGFL7, EFEMP2, SNAI1, COL1A2, COL1A1, MFAP4, FBLN5, COL3A1, ELN, JTB, DHDDS, SEPHS2, CD5, SCART1, CD163L1, CD163, SSC4D and SSC5D.

### Functional Enrichment Analysis of Genes Co-expressed With LOX/LOXL2

Considering the expression level of LOX family in GC tumor tissues and their prognostic values in GC, LOX and LOXL2 were taken into next functional enrichment analysis. The top 50 genes, which had the most significant correlation with LOX/LOXL2 generated by cBioPortal, were included in the following functional enrichment analysis using Metascape.

**FIGURE 6:**
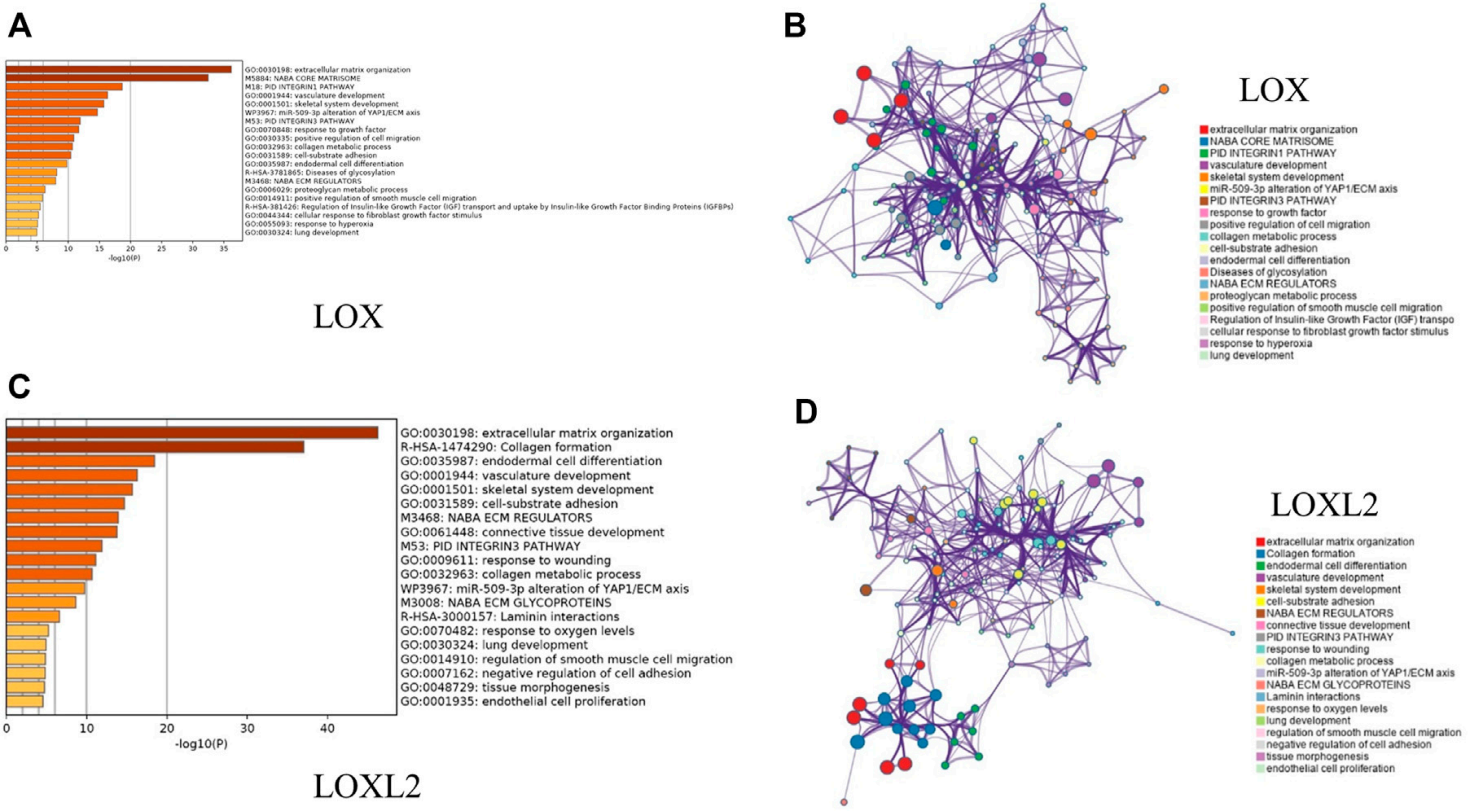
Functional enrichment analysis of genes co-expressed with LOX and LOXL2 using Metascape. **(A**,**C)** Heatmaps of the molecular functions, biological processes, or pathways enriched with LOX and LOXL2 co-expressed genes. The bar color shade was decided by the *p* value, the deeper the shade, the less the *p* value. **(B**,**D)** Networks exhibiting interactions among the clusters of genes enriched in the molecular functions, biological processes, or pathways presented in the heatmaps. The points in different colors represented clusters of genes enriched in different molecular functions, biological processes, and pathways.

The results shown in Figure 6 indicated that the most top 5 significant biological process with LOX and its co-expressed genes were GO: 0030198 (extracellular matrix organization), M5884 (NABA CORE MATRISOME), M18 (PID INTEGRINL PATHWAY), GP: 0001944 (vasculature development), and GO: 0001501 (skeletal system development) (Figure 6A); while the most top 5 with LOXL2 were GO: 0030198 (extracellular matrix organization), R-HAS-1474290 (Collagen formation), GO: 0035987 (endodermal cell differentiation), GP: 0001944 (vasculature development), and GO: 0001501 (skeletal system development) (Figure 6C). Figures 6B,D were networks that exhibited the interactions among cluster of genes enriched in biological processes and pathways mentioned above.

## Discussion

The LOX family has been reported to be dysregulated in a number of cancers (Li et al., 2015; Salvador et al., 2017; Shao et al., 2019; Zeltz et al., 2019; Hu L. et al., 2020). Although the role of LOX family in tumorigenesis and prognosis of several cancers has been partially confirmed, further bioinformatics analysis of GChas yet to be performed. In this study, we used multitalented public databases to reveal the dysregulated expression of the LOX family and their relations with tumor stage and prognosis. We mainly found that the mRNA expression levels of LOX, LOXL1 and LOXL2 were significantly higher in GC, the expression level of LOXL3 was contrary in different databases, while the expression level of LOXL4 made no difference; the expression levels of LOX, LOXL1 and LOXL3 were higher in stages 2–4 than that of normal tissues and stage 1, while the mRNA level of LOXL2 in stage 1–4 was higher than normal tissues; patients with high expression of LOX and LOXL 2-4 had poor OS; the genes correlated with LOXL2/4 were enriched in extracellular matrix organization, vasculature development and skeletal system development.

LOX is a secreted extracellular matrix protein that plays an important role in remodeling the extracellular matrix and promoting tumor progression. Higher LOX mRNA expression was detected in GC tissues than that in adjacent normal gastric tissues, and was significantly correlated with the invasion depth, tumor differentiation, lymph node metastasis, lymphatic invasion, venous invasion, and peritoneal metastasis in GC patients, predicting a poor prognosis of GC patients with high expression of LOX (Zhang et al., 2013). Besides, the mRNA and protein levels of LOX in GC cells and tissues were higher under hypoxia condition than that under normoxia condition (Kasashima et al., 2016). The number of migrating and invading GC cells in hypoxia was significantly decreased after knockdown of LOX (Kasashima et al., 2016). The mechanism involved in LOX-mediated proliferation facilitation in GC is enhancement of Warburg effect through regulation of HIF-1α and c-Myc (Li et al., 2019). The risk of macrophages high density, high microvessel density (MVD), low neomicrovessel maturation, MMP-9 expression and low type IV collagen was elevated after LOX overexpression, suggesting that LOX activated cancer stromal cells and facilitated the progression of GC (Peng et al., 2018). Combining LOX with CEA, CA724, CA199, and CA125 could increase the sensitivity of predicting lymph nodes metastasis and peritoneal metastasis in GC (Lai et al., 2014). Similar results have also been confirmed in our research, however, one study indicated that LOX expression was downregulated in GC, and LOX functioned as a tumor suppressor (Kaneda et al., 2004). Therefore, LOX function in GC needs to be further explored.

Relatively, few data are available on the role of LOXL1 in tumorigenesis. LOXL1 was overexpressed in GC cells, and high LOXL1 expression is a poor prognostic factor in GC patients (Kasashima et al., 2018). Moreover, LOXL1 is associated with peritoneal dissemination, potentially via promoting EMT in GC cells, and high LOXL1 expression was associated with poorly differentiated histological type, lymph node metastasis, and poor prognosis in GC (Hu Q. et al., 2020). Our study also revealed that LOXL1 is highly expressed in GC and may be related to the prognosis, although the results of the two survival databases are inconsistent.

It is first reported that LOXL2 promotes tumor progression and is associated with poor prognosis in breast cancer (Akiri et al., 2003). LOXL2 was overexpressed in GC versus normal tissues, and overexpression of LOXL2 was associated with depth of tumor invasion, lymph node metastasis and poorer overall survival (Peng et al., 2009). Furthermore, secreted LOXL2 promotes GC metastasis via Src kinase/Focal adhesion kinase (Src/FAK) pathway (Peng et al., 2009). LOXL2 expression in stromal cells was significantly associated with tumor invasion depth, lymph node metastasis, lymphatic invasion, venous invasion, peritoneal dissemination, and survival in GC patients (Kasashima et al., 2014). In our report, we illustrated that the expression of LOXL2 in GC tissues was higher than that in normal tissues, and this expression was markedly correlated with tumor stage and poor OS in patients with GC, which was consistent with reports above.

Even though LOXL3 expression was also detected in some kinds of tumors, studies have been conducted on LOXL3 were fewer. The expression of LOXL3 was detected mainly in the nucleus, and the expression of LOXL3 was correlated with tumor invasion, lymph node metastasis, and poorer prognosis of patients (Kasashima et al., 2018). Additionally, TGF-induced LOXL3 upregulation in GC cells, suggesting that LOXL3 was downstream from the TGF-signaling pathway (Kasashima et al., 2018). Our research showed that high expression of LOXL3 was confirmed in GC of TCGA data by using UALCAN database, and the expression was correlated with tumor stage, while there was no difference in the results of Oncomine and GEPIA. The survival analysis also verified that high expression was related to poor prognosis, we speculate that the prognosis of tumors are related to a variety of factors and this may be related to the target of certain drugs in the treatment of gastric cancer. Recently, more studies about LOXL3 have been published, the roles attributed to LOXL3 should be further determined.

LOXL4 was significantly up-regulated in gastric carcinoma tissues, and this over-expression is significantly correlated with tumor size, depth of tumor invasion, lymph node metastasis, higher TNM stages and poor prognosis (Li et al., 2015). LOXL4 may promote proliferation and metastasis via regulate FAK/Src pathway in GC cells (Li et al., 2015). The results about LOXL4 conducted by Kasashima et al. were similar with LOXL3 (Kasashima et al., 2018). In contrast, comprehensive bioinformatics analysis of multiple databases in our study did not yield positive results in expression of LOXL4. In other aspects, survival analysis showed that LOXL4 is associated with poor prognosis, suggesting that LOXL4 is implicated in the progression of GC. This prognostic-related reason may be the same as LOXL3.

In the functional enrichment analysis of genes co-expressed with LOX/LOXL2, the most significant biological process of LOX/LOXL2 and their co-expressed genes is ECM. ECM plays a key role in the occurrence and metastasis of gastric cancer. The destruction of the tightly coordinated ECM tissue will damage the structure and function of the gastric tissue, eventually leading to the progression of gastric cancer (Moreira et al., 2020). We speculate that LOX and LOXL2 affect the occurrence and development of gastric cancer by participating in the regulation of extracellular matrix, but further research is still needed.

Due to the secreted nature of the LOX family members, their detectable presence in the blood, and the well-established correlation between LOX family enzyme expression and prognosis in many cancers, the LOX family offers promise as an inexpensive and non-invasive companion biomarker for cancers (Setargew et al., 2021). The LOX family of enzymes are favorable targets for anti-stromal therapeutics because of their importance in cancer development and progression. A number of studies have examined the use of LOX family inhibitors in cancer therapy (Jiang et al., 2020; Smithen et al., 2020). CCT365623 is a LOX inhibitor based on methylaminothiophene. It has shown that its inhibitory effect can lead to delayed tumor development and reduced lung metastasis in mouse breast cancer models. But it has not yet been tested in a clinical (Smithen et al., 2020). XS-5382A, an oral LOXL2 inhibitor, has been shown to slow tumor growth and reduce collagen accumulation in LY2 oral cancer models and is currently being investigated in Phase 1 clinical trials in healthy adults (Clinical trial identifier: NCT04183517) (Mahjour et al., 2019). Although no inhibitors of the LOX family have currently been approved for routine clinical practice, the developing LOX family inhibitors have shown high specificity and low toxicity.

However, there are limitations in our research. Bioinformatics analysis alone cannot determine the specific mechanism of LOX family in GC. The role of the LOX family in GC might be complex, and more clinical studies and in-depth experiments are needed to verify the diagnostic value of these LOX family and explore the potential mechanism of LOX family affecting the development of GC.

## Conclusion

In this study, through systematically analyzing the expression and prognostic value of LOX family in GC, we indicated that the LOX family, especially LOX and LOXL2, might play an important role in GC oncogenesis, and they may become biomarkers for predicting tumor prognosis and potential targets for tumor therapy.

## Data Availability

The original contributions presented in the study are included in the article/supplementary material, further inquiries can be directed to the corresponding author.
